# Database of epidemic trends and control measures during the first wave of COVID-19 in mainland China

**DOI:** 10.1016/j.ijid.2020.10.075

**Published:** 2021-01

**Authors:** Han Fu, Haowei Wang, Xiaoyue Xi, Adhiratha Boonyasiri, Yuanrong Wang, Wes Hinsley, Keith J. Fraser, Ruth McCabe, Daniela Olivera Mesa, Janetta Skarp, Alice Ledda, Tamsin Dewé, Amy Dighe, Peter Winskill, Sabine L. van Elsland, Kylie E.C. Ainslie, Marc Baguelin, Samir Bhatt, Olivia Boyd, Nicholas F. Brazeau, Lorenzo Cattarino, Giovanni Charles, Helen Coupland, Zulma M. Cucunuba, Gina Cuomo-Dannenburg, Christl A. Donnelly, Ilaria Dorigatti, Oliver D. Eales, Richard G. FitzJohn, Seth Flaxman, Katy A.M. Gaythorpe, Azra C. Ghani, William D. Green, Arran Hamlet, Katharina Hauck, David J. Haw, Benjamin Jeffrey, Daniel J. Laydon, John A. Lees, Thomas Mellan, Swapnil Mishra, Gemma Nedjati-Gilani, Pierre Nouvellet, Lucy Okell, Kris V. Parag, Manon Ragonnet-Cronin, Steven Riley, Nora Schmit, Hayley A. Thompson, H.Juliette T. Unwin, Robert Verity, Michaela A.C. Vollmer, Erik Volz, Patrick G.T. Walker, Caroline E. Walters, Oliver J. Watson, Charles Whittaker, Lilith K. Whittles, Natsuko Imai, Sangeeta Bhatia, Neil M. Ferguson

**Affiliations:** a MRC Centre for Global Infectious Disease Analysis, Abdul Latif Jameel Institute for Disease and Emergency Analytics (J-IDEA), Imperial College London, London, UK; b Department of Mathematics, Imperial College London, London, UK; c NIHR Health Protection Research Unit in Healthcare Associated Infections and Antimicrobial Resistance, Imperial College London, London, UK; d Department of Statistics, University of Oxford, Oxford, UK; e School of Life Sciences, University of Sussex, Brighton, UK

**Keywords:** COVID-19, China, Epidemic, Control measure, Case fatality ratio, Contact

## Abstract

**Objectives:**

In this data collation study, we aimed to provide a comprehensive database describing the epidemic trends and responses during the first wave of coronavirus disease 2019 (COVID-19) throughout the main provinces in China.

**Methods:**

From mid-January to March 2020, we extracted publicly available data regarding the spread and control of COVID-19 from 31 provincial health authorities and major media outlets in mainland China. Based on these data, we conducted descriptive analyses of the epidemic in the six most-affected provinces.

**Results:**

School closures, travel restrictions, community-level lockdown, and contact tracing were introduced concurrently around late January but subsequent epidemic trends differed among provinces. Compared with Hubei, the other five most-affected provinces reported a lower crude case fatality ratio and proportion of critical and severe hospitalised cases. From March 2020, as the local transmission of COVID-19 declined, switching the focus of measures to the testing and quarantine of inbound travellers may have helped to sustain the control of the epidemic.

**Conclusions:**

Aggregated indicators of case notifications and severity distributions are essential for monitoring an epidemic. A publicly available database containing these indicators and information regarding control measures is a useful resource for further research and policy planning in response to the COVID-19 epidemic.

## Highlights

• COVID-19 measures were applied on similar dates in provinces throughout China.
• Disease severity was much greater in Hubei compared with other provinces.
• Provincial data on epidemics and interventions is available for further research.

## Introduction

The coronavirus disease 2019 (COVID-19) outbreak was first reported in Wuhan City, Hubei Province, China in late December 2019 (Xinhua, 2020b). From late January 2020, many provinces in China began to report confirmed COVID-19 cases. Stringent social distancing, travel restrictions, contact tracing, environmental disinfection, and other strategies were implemented to control the epidemic. From late February 2020, other countries reported rising numbers of infections, whereas a declining epidemic trend was observed in China. Considering the global spread of the pathogen, the World Health Organization (2020) declared COVID-19 a pandemic on 11 March 2020. Although an increase was observed in the number of confirmed cases after June 2020, the overall size of the epidemic remained small in China.

The Imperial College London COVID-19 Response Team initiated data collection activities in mid-January 2020 to understand the epidemic in China. Together with volunteers, the Imperial Team made considerable efforts to collate aggregated data and individual patient information from publicly available, national, and local situation reports published by health authorities in China. Individual case or death reports are considered crucial for informing the determinants of disease severity and fatality during the emergence of the epidemic (Verity et al., 2020), but the reporting was scattered and unfeasible when new cases increased exponentially. Alternatively, aggregated notifications of cases and contacts were more accessible and mostly recorded in a standardised format across provinces in China. In addition to these indicators derived from the surveillance system, empirical experience from the implementation of control measures was essential for interpreting the variations related to the epidemic trends in the context.

The data we extracted from Chinese official reports may also be useful for the wider research community by building on other existing data collection activities (Xu et al., 2020; Zhang et al., 2020). In the present study, we collated the data and obtained an overview of the epidemic trends and control measures in China based on descriptive analyses. These exploratory findings highlight the potential applications of the database and provide insights into the epidemic response in other countries.

## Methods

### Data collation

Daily situation reports of the COVID-19 epidemic were extracted from mid-January to 31 March 2020 in 31 provinces/municipalities (with equivalent levels of administration) of mainland China. We downloaded these reports from the websites of local health commissions and used Google translate to obtain English versions for each province/municipality. In addition, reports from the National Health Commission website and Wuhan City Health Commission website were included. We extracted aggregated numbers of cases, deaths, recoveries, contacts, and details on disease severity and case importation from the official reports released each day (Table 1). The quantitative results for each province/municipality were extracted into a spreadsheet. Each record entry was independently checked and compared with the original situation reports by a second researcher. Both the spreadsheet and original situation reports are available at Github: https://github.com/mrc-ide/covid19_mainland_China_report.

**Table 1:** Aggregated numbers extracted from provincial/municipal reports in mainland China.

Variables^a^	Definition/description^b^
Cumulative cases	Number of total confirmed cases by the end of the reporting date
Cumulative imported cases	Number of total confirmed cases imported from other countries by the end of the reporting date
Cumulative recoveries	Number of total cases discharged after recovery by the end of the reporting date
Cumulative deaths	Number of total deaths by the end of the reporting date
Cumulative close contacts	Number of total close contacts by the end of the reporting date
Cumulative close contacts completing quarantine	Number of total close contacts who completed 14-day quarantine by the end of the reporting date
Current cases	Number of confirmed cases currently hospitalised on the reporting date
Current critical and severe cases	Number of critical and severe cases currently hospitalised on the reporting date
Current close contacts under quarantine	Number of contacts currently under quarantine (medical observation) on the reporting date

a Only variables used in this descriptive analysis are listed. A full list of extracted variables can be found in the data dictionary of the GitHub repository mentioned earlier. Newly reported numbers were derived by taking the difference in the cumulative numbers between two consecutive reporting dates.

b Case definitions and clinical severity from the guidelines of the National Health Commission were used (National Health Commission of the People's Republic of China, 2020b).

We reviewed the timing of implementation and subsequent lifting of the following control measures: i) cancellation of cross-province public transportation; ii) temperature checks for inbound travellers at provincial borders; and iii) community-level lockdown (so-called ‘closed-off management’, including measures such as shop closures and banning non-resident entry (Zhu, 2020). We searched official notices and announcements published by the national and provincial/municipal governments as well as local news for information regarding these non-pharmaceutical interventions. The closure and reopening dates of primary, middle, and high schools, and universities were also extracted. In addition, we monitored the progress of economic activity resumption through the reopening of ‘designated enterprises’, which comprised registered companies with an annual revenue exceeding 2.8 million United States Dollars (20 million Chinese Yuan) (National Bureau of Statistics of China, 2018).

### Descriptive analysis of epidemic trends

**Figure 1:**
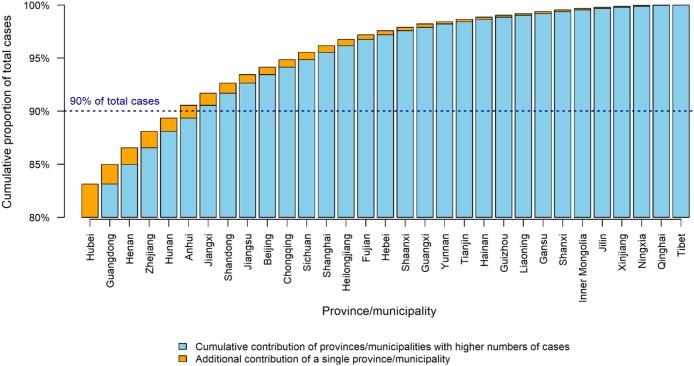
Cumulative proportions of total cases contributed by province/municipality up to 31 March 2020. Thirty-one provinces/municipalities in China ranked in descending order (left to right) of total confirmed cases up to 31 March. Yellow bars represent the proportion of national confirmed cases contributed by a single province/municipality. Blue bars are the cumulative contributions from provinces/municipalities with higher numbers of reported cases.

Based on the aggregated data collated for each province/municipality, we conducted a descriptive analysis to understand the epidemic trends and their possible associations with the interventions implemented. We focused on the ‘six provinces’ (Hubei, Guangdong, Henan, Zhejiang, Hunan, and Anhui) that reported the highest numbers of confirmed cases up to the end of March 2020. These provinces accounted for 90% of the total COVID-19 cases in mainland China. Hubei alone accounted for 80% of the total number of reported cases (Figure 1).

We first calculated the proportion of recoveries from 15 January to 31 March as: $(\frac{number\;of\;cumulative\;\;recoveries}{number\;of\;cumulative\;\;confirmed\;\;cases})\times 100\%$. It should be noted that almost all of the confirmed cases were hospitalised for isolation and medical care in mainland China, and hospitals were responsible for reporting cases to the surveillance system. Recoveries in this setting were defined as hospitalised cases who met the criteria for discharge, including symptom relief and negative test results (National Health Commission of the People's Republic of China, 2020b).

To investigate the disease severity across provinces, we obtained the crude case-fatality ratio (cCFR) as: $\;\left(\frac{number\;of\;cumulative\;deaths}{number\;of\;cumulative\;confirmed\;cases}\right)\times 100\text{\%}.$ Confidence intervals (CI) were calculated for the cCFRs based on binomial distributions, with an underlying assumption that all cases with unresolved outcomes would eventually recover. The cCFR may be biased at reflecting the disease severity due to under-ascertainment and delays in death reporting (Garske et al., 2009), but it may be an approximate estimate near the end of an epidemic when the capacity for case detection improves and the outcomes of cases are mostly known. We also captured the varying need for critical care over time using the distribution of case severity among currently hospitalised cases as: $\;\left(\frac{number\;of\;critical\;or\;severe\;current\;cases}{number\;of\;current\;cases}\right)\times 100\%$.

According to the guidelines for contact investigation published by the Chinese Center for Disease Control and Prevention (2020), those who have close contact with a confirmed case of COVID-19 during the period from two days prior to their symptom onset to isolation should be quarantined at home or a specific facility for 14 days. To assess the scale and effort involved in contact tracing across provinces, we calculated the contact-to-case ratio as: $\left(\frac{number\;of\;contacts}{number\;of\;confirmed\;cases}\right)$. In this analysis, the contact-to-case ratio was first calculated based on the cumulative numbers up to 31 March for each of the six provinces. We then derived the same ratio by taking the newly reported numbers at the national level over the observation period. The guidelines also recommended that epidemiological surveys of the contact history of new cases should be completed within 24 hours from case confirmation, so we conducted an alternative analysis by assuming a 1-day lag between the reporting of cases and contacts.

## Results

### Overview of COVID-19 control measures

**Figure 2:**
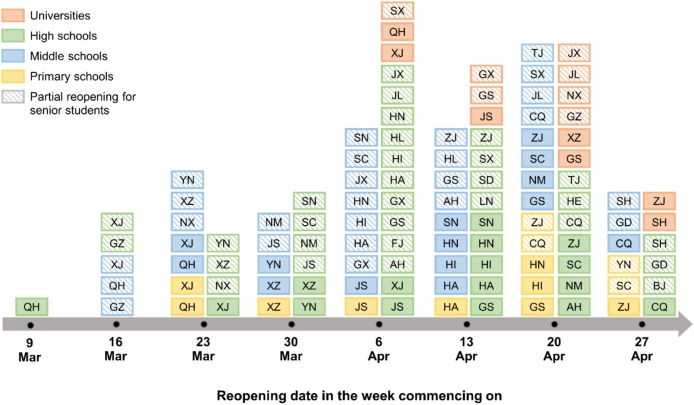
Reopening dates for primary, middle, and high schools, and universities. Dates of school reopening are summarised weekly for March and April 2020. Each rectangle indicates the reopening of a specific level of school (denoted by colours) in a province/municipality (denoted by text abbreviations). Rectangles fully filled with colour represent reopening at full scale, whereas those filled with diagonal lines represent partial reopening for senior or research students. Reopening dates were extracted from official announcements and local news (available at Github: https://github.com/mrc-ide/covid19_mainland_China_report). Abbreviations for provinces/municipalities: AH – Anhui, BJ – Beijing, CQ – Chongqing, FJ – Fujian, GD – Guangdong, GS – Gansu, GZ – Guizhou, GX – Guangxi, HA – Henan, HE – Hebei, HI – Hainan, HL – Heilongjiang, HN – Hunan, JS – Jiangsu, JL – Jilin, JX – Jiangxi, LN – Liaoning, NM – Inner Mongolia, NX – Ningxia, QH – Qinghai, SC – Sichuan, SD – Shandong, SH – Shanghai, SN – Shaanxi, SX – Shanxi, TJ – Tianjin, XJ – Xinjiang, XZ – Tibet, YN – Yunan, and ZJ – Zhejiang.

On 26 January 2020, The State Council of The People's Republic of China (2020a) announced the extension of the school winter vacation with the aim of COVID-19 control, where the schools had been closed since 24 January or earlier for the Chinese New Year. The reopening of schools was postponed multiple times for epidemic control and prevention (China Central Television, 2020a, China Central Television, 2020b). In late March, local governments in provinces affected less by COVID-19 began to reopen their schools, particularly for senior-year students in middle and high schools (Figure 2). Most of the other provinces/municipalities kept their schools closed until late April and May. Due to the interruption of education, the National College Entrance Examination in China was postponed by one month until July 2020 (Hubei Province COVID-19 Prevention and Control Centre, 2020a). Reopening of universities was generally further delayed because it involves large-scale, cross-province population movement (China Central Television, 2020a).

**Figure 3:**
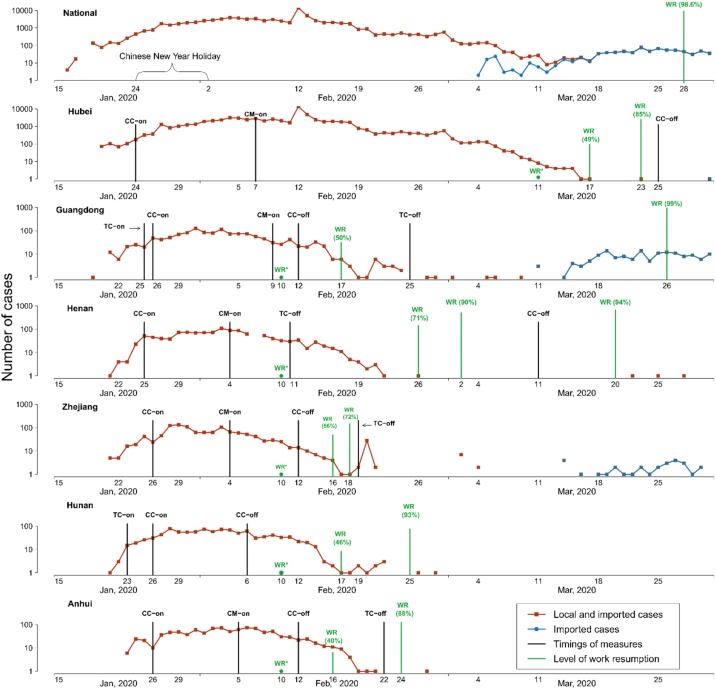
Newly confirmed cases and timings of control measures by province. Numbers of confirmed cases at national and provincial levels are shown on a log scale. Vertical lines denote the timings of implementing and relaxing control measures (black), and related work resumption statistics (green). The asterisks (*) indicate the initiation date for work resumption. Abbreviations for control measures: CC – cancellation of cross-border public transportation, TC – temperature checks at provincial borders, and CM – closed-off management at community level.

In addition to school closures, the State Council (2020a) extended the end of the national Spring holiday (Chinese New Year holiday) from 30 January to 2 February 2020. In response to the COVID-19 epidemic, Beijing, Shanghai, and several local governments further delayed the resumption of work until 10 February (The State Council of The People's Republic of China, 2020b). In the most severely affected province in China, Hubei, the date for returning to work was first postponed until 14 February (The State Council of The People's Republic of China, 2020b) and then until 11 March (Hubei Province COVID-19 Prevention and Control Centre, 2020b). In Wuhan City, general industries (except for essential service providers and key global enterprises) did not resume operation until 21 March (Chutian Metropolis Daily, 2020). In accordance with the national guideline to prioritise industries that provide essential products and services (Xinhua, 2020a), production and transportation were restored in stages. At the end of March, most of the provinces/municipalities in China reported a high degree of recovery in terms of their economic activities, where more than 90% of the ‘designated enterprises’ had returned to business (Figure 3). Work activities resumed much later in Hubei than other provinces/municipalities, but it was reported that as many as 85% of the local ‘designated enterprises’ had resumed operation by 23 March (Chutian Metropolis Daily, 2020).

In addition to the key dates for initiating and lifting the three most common control measures, Figure 3 shows the daily confirmed cases over time in the six provinces in China. Measures related to provincial border control comprising cancellation of cross-province public transportation and temperature checks for inbound travellers at provincial borders were consistently imposed in late January across provinces. However, at the time when these measures were introduced, more than 700 cumulative cases were reported in Hubei but less than 150 cases were observed in the other provinces where the local epidemic was at an earlier stage. The general implementation of community-level closed-off management was introduced during the peak of the local epidemic. Relaxation of these measures varied by province but it was related to the decline in daily confirmed cases. Except for Hubei, the local epidemic in the other five provinces was mostly suppressed in late February. Zhejiang and Guangdong provinces, where international airports are located, reported a second wave of COVID-19 driven by incoming travellers from the beginning of March. However, the caseload caused by this second wave was much smaller than the first because measures to stop secondary transmission were implemented at the border for inbound passengers (Zhang, 2020).

### Descriptive analysis of COVID-19 epidemics in the six provinces with the highest total caseloads

**Figure 4:**
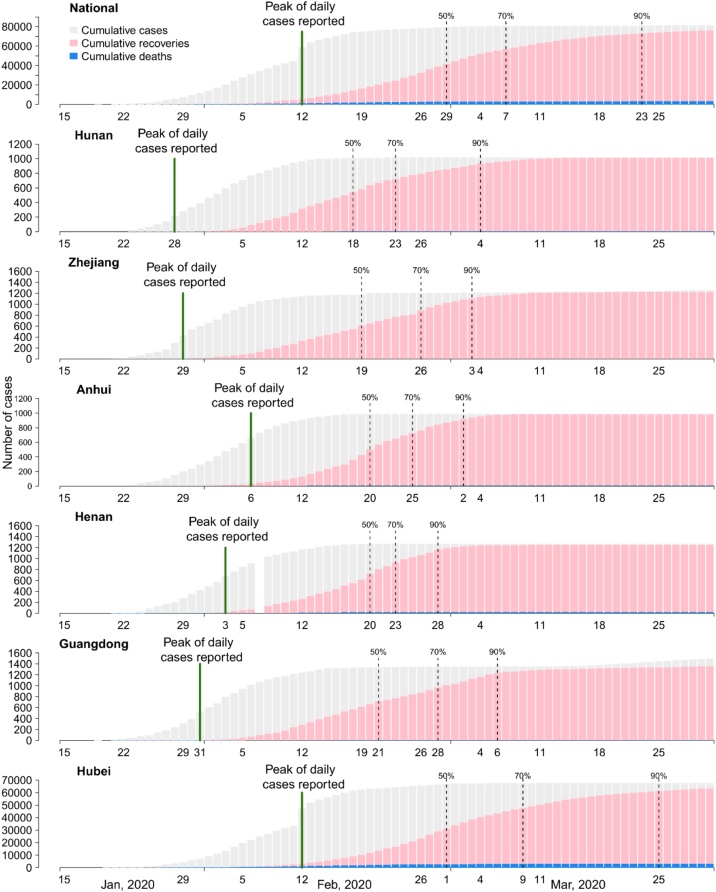
Cumulative cases, deaths, and recoveries by province. Bars represent the cumulative numbers of cases (grey), recoveries (pink), and deaths (blue). Black vertical dashed lines show the dates when 50%, 70%, and 90% of recoveries among all cases were reached. Green vertical solid lines show the dates when the peak number of daily confirmed cases occurred. The six provinces are ranked from top to down by the date when 50% recovery was achieved. It should be noted the range for the y-axis differs among provinces to fit the magnitude of cases.

We explored the association between the epidemic trend and health care burden based on the time-varying proportions of the total confirmed cases who recovered (Figure 4). Most provinces reported 50% recovery by mid-February at 2–3 weeks after the peak of daily confirmed cases in late January or early February. The national trend was delayed by approximate 10 days due to the severe epidemic in Hubei, where the peak of daily cases and 50% recovery occurred on 12 and 29 February, respectively. From 50% to 90% recovery, the duration was longer in Guangdong and Hubei compared with the other provinces because newly reported cases continued to increase in late February and early March.

A wide variation in cCFRs was reported in each province by 31 March 2020 (Table 2). Most of the other provinces had a cCFR less than 1% but Hubei had a cCFR of 4.71% (95% CI = 4.55–4.87%). In addition, Henan province had the second-highest cCFR (1.73%, 95% CI = 1.09–2.61%) among the six provinces analysed. The most affected areas in Henan province comprising Xinyang City, Nanyang City, and Zhumadian City are adjacent to Hubei province, and many workers returned from Wuhan before the lockdown due to the Chinese New Year holiday (Su and Song, 2020). Thus, both the geographical and social connections with Hubei may explain the stronger impact of the COVID-19 epidemic in Henan.

**Table 2:** Crude case-fatality ratios up to 31 March by province.

Province	Total cases	Total deaths	Total recoveries	Percentage of cases without a resolved outcome	cCFR^a^ (95%CI)
National	81,554	3,312	76,238	2.46%	4.06% (3.93%, 4.20%)
Hubei	67,802	3,193	63,326	1.89%	4.71% (4.55%, 4.87%)
Guangdong	1,501	8	1,357	9.06%	0.53% (0.23%, 1.05%)
Henan	1,273	22	1,250	0.08%	1.73% (1.09%, 2.61%)
Zhejiang	1,257	1	1,226	2.39%	0.08% (<0.01%, 0.44%)
Hunan	1,018	4	1,014	0%	0.39% (0.11%, 1.01%)
Anhui	990	6	984	0%	0.61% (0.22%, 1.13%)

a Crude case-fatality ratios (cCFRs) were calculated as the proportion of cumulative deaths among confirmed cases and their 95% confidence intervals (CIs) were obtained based on an assumption of a binomial distribution.

**Figure 5:**
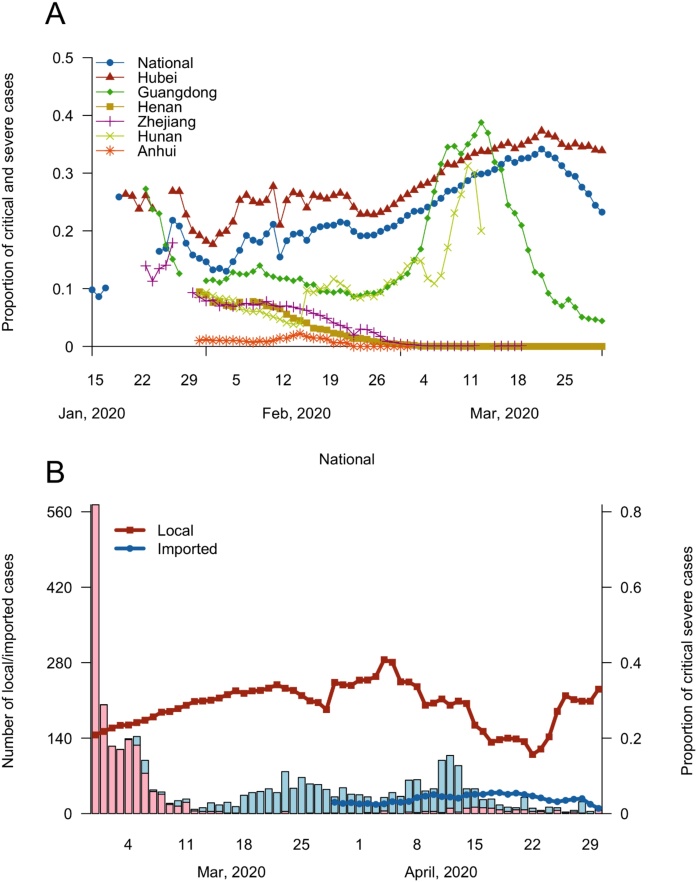
Severity of COVID-19 among current cases (A) by province and (B) by locally transmitted and imported cases. Proportions of critical and severe cases among all current cases are presented in the upper panel (A) by national and six provinces with the highest caseloads from 15 January to 31 March. In the lower panel (B), disease severity is shown according to locally transmitted (red) and imported (blue) cases from 1 March to 30 April. Solid lines represent the proportions of critical and severe cases at a level corresponding to the right y-axis, and bars denote the absolute numbers of total cases with the scale denoted on the left y-axis.

Provincial differences in disease severity were also demonstrated by the analysis of hospitalised cases (Figure 5), where Hubei reported a particularly high proportion of critical and severe cases (20–30%). In terms of the temporal trend, a tendency to capture cases with more serious symptoms was found consistently across the six provinces with the highest caseloads in early February (Figure 5A). In March, the proportion of critical or severe cases increased again whereas the total numbers of hospitalised cases declined, thereby reflecting the longer hospitalisation period for severe cases compared with mild cases. However, a distinct trend was seen in Guangdong from mid-March, with a sharp decline in the proportion of critical or severe cases. This decline coincided with the increase in cases imported from foreign countries, where these cases tended to have mild symptoms (Figure 5B).

**Figure 6:**
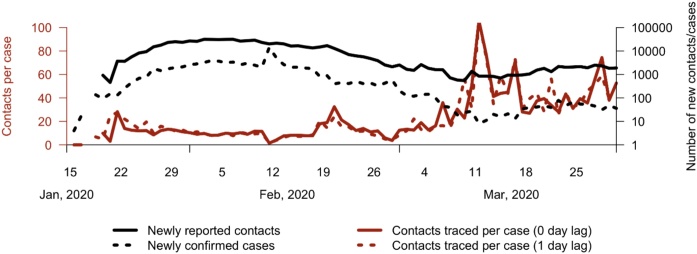
Contacts traced per newly confirmed case. The trends in contacts traced per newly confirmed case are presented by assuming 0 (red solid line) and 1 (red dashed line) day lags based on the y-axis shown on the left-hand side. Numbers of daily contacts and cases are shown on a log scale by black solid and black dashed lines, respectively, and they correspond to the y-axis on the right-hand side.

Finally, we investigated the scale of contact tracing for infection control at the national and provincial levels by using the ratio of total contacts relative to total cases by the end of March (Table 3). On average, 20–40 close contacts were traced per confirmed case. Hubei province reported a particularly low contact-to-case ratio compared with other provinces. To further explore the change in the number of contacts traced during the epidemic, we again calculated the contact-to-case ratio based on the daily numbers of reported contacts and confirmed cases (Figure 6). Less than 20 contacts were traced for each new case during most of January and February, but the contact-to-case ratio increased in March. This increase in the ratio was caused by the greater number of total contacts reported in provinces outside Hubei, probably due to imported cases. In the exploratory scenario that considered the 1-day delay in contact tracing following case confirmation, the general trend in the contact-to-case ratio over time was consistent with the scenario without considering a delay.

**Table 3:** Average number of contacts traced per confirmed cases by province.

Province	Total contacts	Total cases	Contact-to-case ratios^a^
National	707,913	81,554	8.68
Hubei	278,179	67,802	4.10
Guangdong^b^	–	1,501	–
Henan	40,019	1,273	31.44
Zhejiang	46,764	1,257	37.20
Hunan	27,331	1,018	26.85
Anhui	28,981	990	29.27

a Cumulative numbers of confirmed cases and contacts reported by 31 March 2020 were used to calculate contact-to-case ratios.

b Numbers of total contacts were not reported in Guangdong, and thus the contact-to-case ratio is not available.

## Discussion

In this study, we conducted data collation and descriptive analyses of the COVID-19 epidemic trends and control measures in mainland China between mid-January and March 2020. In most provinces, the local epidemics peaked in early February and declined in early March, but they were not completely eliminated because the number of imported cases increased. School closures, travel restrictions, contact tracing, and other control measures were enforced at a similar time from late January across provinces. However, in Hubei where the origin of the COVID-19 pandemic was reported, increased levels of case fatality and severity were reported compared with the other five most badly affected provinces analysed. Based on the descriptions of the epidemic trends and timings of interventions, we observed the potential effects of early implementation and timely adjustment of the control strategies on containing the COVID-19 epidemic in mainland China. The collated data have been made available and they may provide a useful resource for further research into epidemic control and policy planning.

Domestic travel restrictions were implemented at similar times across provinces in China (Figure 3), although the epidemic situation differed in each province. These restrictions were introduced for provinces not neighbouring Hubei when few cumulative cases were reported, and thus they may have been more effective at limiting and averting transmission. Lifting travel restrictions depended on the control of local epidemics but also the risk of case importation and transmission. For example, on 11 March, Henan province restored both cross-province and inner-province public transportation, except for routes connected with adjacent Hubei (Henan Daily, 2020). The implementation of case-based measures such as community-level lockdown (closed-off management) and contact tracing was initiated after the notification of confirmed cases and the detection of outbreak clusters in each province.

The timing of school reopening in China depended on the local epidemic situation (China Central Television, 2020b), but the general strategy was shared across multiple provinces (Figure 3). Staged reopening was widely observed, where it was suggested that senior students in middle and high schools return first, with junior students and elementary schools following one week later. In terms of returning to work, most provinces rapidly resumed their business activities after relaxing constraints on travel and commuting (Figure 4). However, this rapid resumption occurred in the reopened ‘designated enterprises’, which excluded enterprises not considered key industries or smaller scale enterprises. Additional surveys of detailed indicators of resumption, such as the production capacity and attendance of employees, as well as the resumption of other aspects of economic activities will be useful for fully understanding the progress of restoration and inequality in the impacts of COVID-19.

A particularly high cCFR was reported in Hubei compared with the other provinces according to our analysis. This difference in disease severity was also consistently demonstrated by the proportion of critical and severe cases over the last few months. Another study that accounted for right-censoring when estimating the true case fatality demonstrated a similar discrepancy across provinces to that we determined based on the cCFRs (Deng et al., 2020). This similarity also supports the use of cCFRs extracted near the end of the epidemic because these crude proportions were more stabilised (Figure S1) and closer to the true burden (Garske et al., 2009). The severe burden was potentially driven by the explosive increase in cases that overwhelmed local healthcare services during the peak of epidemic, or the varying capacity for case detection over time. In addition, the quality of reporting may have affected the trends in these indicators. For example, on 17 April, the Hubei Health Commission announced corrections to the numbers of deaths and recoveries during the epidemic (National Health Commission of the People's Republic of China, 2020a), which resulted in a further increase in the provincial cCFR from 4.71% to 6.62% (95% CI = 6.44–6.81%) by the end of April.

The indicators comprising cCFR and proportions of currently critical and severe cases exhibited different relationships across the provinces in China (Table 2 & Figure 4). Henan province reported a moderate cCFR but a low proportion of severe cases, whereas an opposite trend in terms of case fatality and severity was observed in Guangdong province. However, interpreting differences in fatality and severity is challenging because these indicators together with the proportion of recovery (Figure 5) reflect the combined effects of different epidemic burdens and local health systems. Collecting further data and assessing the capacities of hospitals for testing, caring, and reporting in different settings will be essential for understanding the factors that determined the COVID-19 burden.

Contact tracing was implemented nationally at the beginning of the epidemic in China as a key strategy coupled with case management (Li et al., 2020). We found that for every confirmed case, four contacts were traced on average in Hubei, whereas over 20 contacts per case were traced in other provinces (Table 3). These numbers of contacts are consistent with the average number of daily contacts according to diary-based contact surveys in Wuhan City and Shanghai. Approximately two and 17 daily contacts were reported for each citizen during the lockdown and before the COVID-19 epidemic, respectively (Zhang et al., 2020), thereby suggesting that stringent social distancing policies could modify the contact patterns and reduce the number of contacts. The overall case burden in each province may also have affected the number of contacts that could be traced by the local public health authority. From early March, the number of contacts traced per case increased, possibly due to large clusters of contacts who shared the same flights and trains among the imported cases who travelled from foreign countries. However, we cannot exclude the possibility that the increased contact-to-case ratio was due to increased investment in personnel training and the establishment of appropriate management systems for contact tracing. These resources could have gradually released other control measures and relieved the epidemic burden. It is uncertain how many contacts were eventually confirmed as cases in most provinces. Further data collation and investigation will allow the effectiveness of contact tracing at reducing COVID-19 transmission to be assessed.

A major limitation of our descriptive analysis is the use of aggregate cases, deaths, recoveries, and contacts data. These indicators are convenient for monitoring and comparing the epidemic trends by province, but further inferring the effects of risk factors on the transmission dynamics is not possible. Patient characteristics such as age and comorbidities are essential for understanding the heterogeneity of disease severity. Estimations of the setting-specific incubation period, reporting delay, and disease progress also rely on the date of symptom onset and care-seeking pathways for individual cases (Zhang et al., 2020). Another limitation was the inability to validate, quantify, and distinguish the impacts of different control measures. Comparisons across provinces only allowed us to explore the temporal relationships between interventions and epidemic trends. Applying dynamic modelling techniques and incorporating additional data sources may advance our understanding of the contributions of different interventions over the course of the epidemic (Flaxman et al., 2020, Lai et al., 2020).

Many containment measures have been implemented in the different provinces of China since the beginning of the COVID-19 outbreak in Wuhan City, Hubei Province. Similar control measures were also introduced in other countries, such as Singapore and South Korea, although the practical implementations varied (Imai et al., 2020). Social distancing and contact tracing measures probably contributed to the reductions in COVID-19 transmission because the reported sizes of the epidemics were relatively small in these countries, as also indicated by a modelling study based on counterfactual scenarios in Chinese provinces (Lai et al., 2020). The driving force of the COVID-19 epidemic in mainland China has shifted from local transmission to importation from other affected countries, so the focus of the response has been modified, such as compulsory testing and quarantine for all incoming travellers (Cui, 2020), and close monitoring of asymptomatic infections (National Health Commission of the People's Republic of China, 2020c). The timely adjustment of control strategies based on surveillance may have contributed to the sustained control of COVID-19. Our review of control measures and analysis of aggregated indicators provides a compact summary of the COVID-19 epidemic across the provinces in mainland China. We demonstrated the contribution of data collation activities and how this publicly available database can be useful for exploring different aspects of COVID-19 control strategies.

## Author contributions

HF, HW, XX, NI, and SB conceptualised and conducted the analysis. HF, HW, XX, AB, YW, SLvE, and NI set up the data collation framework and maintained the quality of the data sets. HF, HW, XX, AB, YW, WH, KJF, RM, DOM, JS, AL, TD, AD, KECA, LC, GC, ODE, NS, HAT, HJTU, and LKW extracted the data. HF wrote the first draft of the manuscript. All authors contributed to the revised version of the manuscript and agreed with its submission.

## Funding

We acknowledge Joint Centre Funding from the UK Medical Research Council and Department for International Development [grant number MR/R015600/1]. This study was also supported by the National Institute for Health Research Health Protection Research Unit in Modelling Methodology, the Abdul Latif Jameel Foundation, and the EDCTP2 programme supported by the 10.13039/501100000780European Union.

## Conflict of interest

The authors declare that they have no conflicts of interest.

## Ethical approval

This study did not require ethical approval because aggregated publicly available data were used.
