# Supplementary material for: Database of epidemic trends and control measures during the first wave of COVID-19 in mainland China
Source: Int J Infect Dis. 2021 Jan;102:463–71. doi: 10.1016/j.ijid.2020.10.075 (PMC7603985; doi:10.1016/j.ijid.2020.10.075)
Supplement: Supplementary file 1 [file mmc1.pdf]

Supplementary material

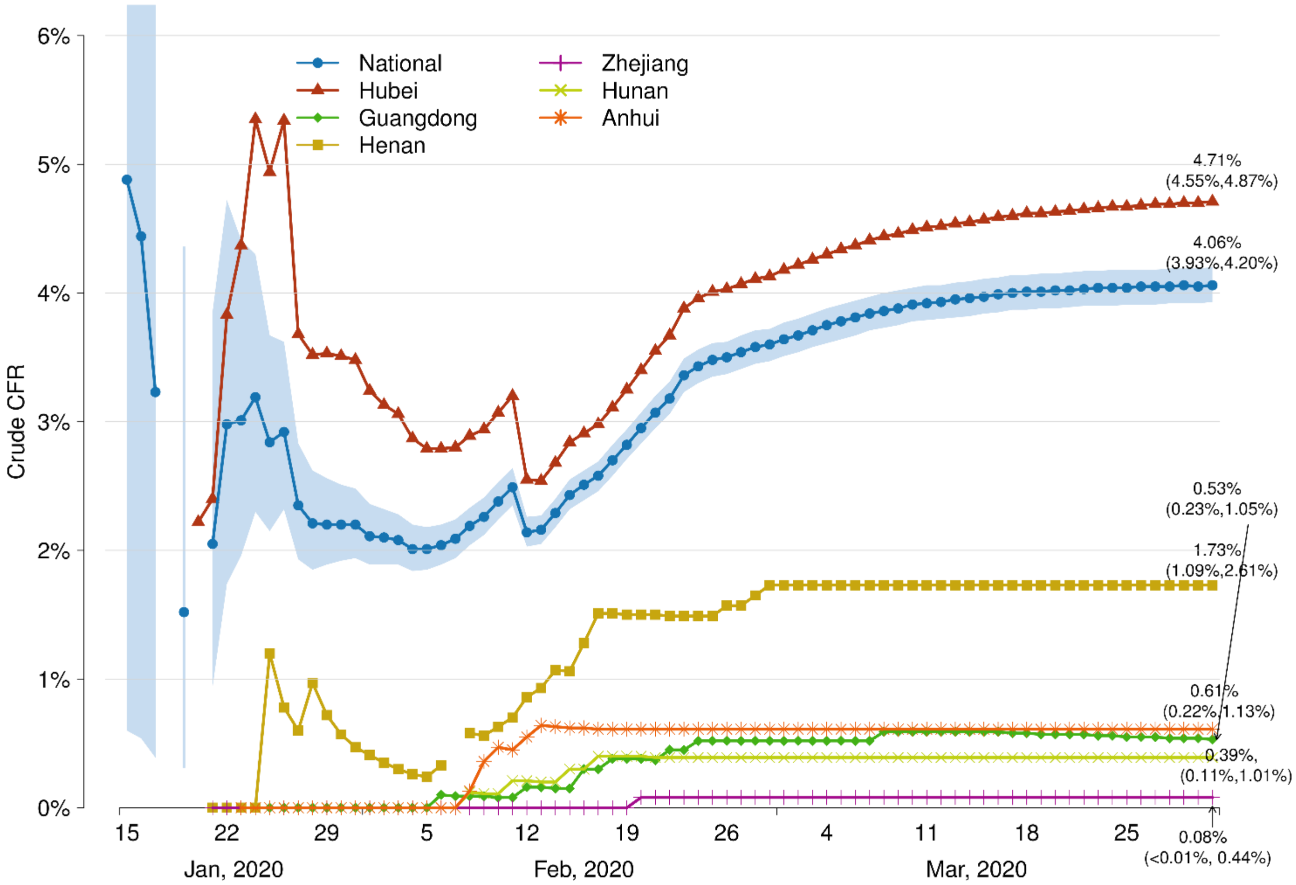

Figure S1. Crude case-fatality ratios (cCFRs) calculated by different dates in the six provinces with the highest total caseloads.

By each date, the proportion of cumulative deaths among cumulative confirmed cases is calculated. Trends of cCFRs are presented in solid lines by province. Numbers at the end of each curve represent the cCFRs by 31 March 2020, with 95% confidence intervals (CIs) showing in brackets. For clarity of presentation, only the time-varying 95%CIs for national cCFRs are shown.
